# Energy Balance-Related Behavior Risk Pattern and Its Correlates During COVID-19 Related Home Confinement

**DOI:** 10.3389/fnut.2021.680105

**Published:** 2021-06-08

**Authors:** Surabhi Bhutani, Michelle R. vanDellen, LeeAnn B. Haskins, Jamie A. Cooper

**Affiliations:** ^1^School of Exercise and Nutritional Sciences, San Diego State University, San Diego, CA, United States; ^2^Department of Psychology, University of Georgia, Athens, GA, United States; ^3^Department of Foods and Nutrition, University of Georgia, Athens, GA, United States

**Keywords:** COVID-19, energy balance, eating behaviors, physical activity, psychological factors

## Abstract

Self-reported weight gain during the COVID-19 shelter-at-home has raised concerns for weight increases as the pandemic continues. We aimed to investigate the relationship of psychological and health markers with energy balance-related behaviors during the pandemic-related extended home confinement. Ratings for stress, boredom, cravings, sleep, self-control, and beliefs about weight control were collected from 1,609 adults using a questionnaire between April 24th–May 4th, 2020, while COVID-19 associated shelter-in-place guidelines were instituted across the US. We calculated four energy balance behavior scores (physical activity risk index, unhealthy eating risk index, healthy eating risk index, sedentary behavior index), and conducted a latent profile analysis of the risk factors. We examined psychological and health correlates of these risk patterns. Boredom, cravings for sweet/savory foods, and high sleepiness ratings related to high risk of increasing unhealthy eating and sedentary behavior and decreasing physical activity and healthy eating. Having greater self-control, control over cravings, or positive mood was related to lowering all aspects of energy intake and energy expenditure risks. Although individuals in risk pattern classes showed similarity in physical activity and healthy/unhealthy eating habits, they exhibited different patterns of positive mood, craving control, food cravings, boredom, and self-control. Psychological and health variables may have a significant role to play in risk behaviors associated with weight gain during the COVID-19 related home confinement. Emerging behavioral patterns may be meaningful in developing targeted weight management interventions during the current pandemic.

## Introduction

In March 2020, the novel severe acute respiratory distress coronavirus 2 (SARS-CoV-2) infection emerged as a global COVID-19 pandemic. As a consequence, widespread shelter-at-home was implemented in the US to prevent the spread of this infection, primarily between March 15th and May 7th, 2020. This public health action markedly disrupted everyday activities and increased unstructured time for people, making weight management a concern (1–3) frequently referred to on social media as “Quarantine 15,” “gaining the COVID-19,” or “fattening the curve.” Indeed, we and others recently showed that 19–28% of adults self-reported gaining 5–10 pounds of body weight during the self-quarantine (3–5). These self-reported weight increases are of concern because literature on holiday weight gain suggests that fluctuations in body weight in a relatively short period can become permanent and lead to a substantial weight gain over decades (6–9). Thus, it is imperative to understand *for whom* self-reported changes in energy balance behaviors categories have a potential to contribute to weight increases during the brief period of lockdown, mainly healthy and unhealthy eating, and physical and sedentary activities.

With shelter-at-home restrictions and inability to practice normal life, numerous possible challenges can affect energy intake and energy expenditure, the two components of energy balance. With regards to energy intake behaviors, COVID-19 disruptions may have introduced multiple influences on people's dietary behaviors which may have produced increased unhealthy eating and/or healthy eating. In particular, during the lockdown people had easy access to snacks and craving inducing energy dense convenience foods (10, 11) and showed greater interest in cooking/baking high-calorie foods (12). Stockpiling and consumption of shelf stable ultra-processed food intake was also frequent (13, 14). Interestingly, increased intake of healthy foods was also reported by many adults (5, 11), possibly due to greater opportunities for cooking at home and a decline in intake if restaurant meals. Attributed to social isolation and restrictions, a decline in physical activity and greater engagement in sedentary behavior, such as increased screen time, has also been reported (11, 15–17). Considering that two-thirds of the US adult population is overweight or obese, it is critical to understand the impact of COVID-19 on energy balance-related behaviors and identify which individuals are most susceptible to altering these behaviors.

COVID-19 lockdown and related social distancing drastically impacted the life of people in the US. People lost their jobs and shifted to work from home schedule while actively taking care of family and dealing with the fear of infection. Travel, social life, and leisure activities were also severely restricted, unlike prior to pandemic. These major life adjustments were accompanied by severe physiological and psychological costs, as reported in multiple studies. In particular, the recent lockdown caused dramatic increases in these state-like psychological variables, such stress, anxiety, low-sleep quality etc. (18–21). Boredom is another psychological consequence of the interruption to work and social routines, which was evident with SARS outbreak related quarantine in 2003 (22), and possibly with the current lockdown. These state-like psychological variables have been known to correlate with greater energy intake (23), more screen time, and low energy expenditure (24). Similarly, stress (25) and high sleepiness (26) are known to promoting cravings for energy-dense foods. Since these state-like psychological variables relate to energy balance behaviors, we expected they might be relevant to explore during COVID-19 lockdown.

With regards to the trait-like psychological variables, some of these factors are known to be known to be protective toward these extreme behavioral alterations. For example, lack of self-control (27, 28) and a lack of belief that body weight can be personally controlled (29, 30) are both part of people's motivational systems and influence self-regulatory processes and goal achievement. Not surprisingly, both are also related to food consumption and other weight management behaviors. Therefore, having these psychological traits may counter the possible negative impact of shelter-at-home on energy intake and expenditure behaviors, and adherence to a healthy and active lifestyle requires self-control and beliefs that body weight can be personally controlled (31). While we recently show self-reported shifts in energy intake and energy expenditure behaviors during the COVID-19 shelter-in-place period using cross-sectional survey data (11), whether these trait-like psychological factors will have a similar protective affect toward energy balance-related behaviors during the COVID-19 lockdown, is of great importance.

Overall, this study aimed to investigate the relationship between relevant demographic characteristics, state- and trait-like psychological markers and energy balance-related behaviors, during the pandemic-related shelter-in-place. Specifically, we examined associations between stress, boredom, cravings, sleep, self-control, BMI, and beliefs about weight control. In addition, we evaluated differences in risk behaviors between demographic groups. Using a Latent Profile Analysis, we also aimed to identify and characterize patterns of health behavior change during the pandemic. We hypothesized that sleep time and quality, craving control, self-control, and beliefs that one can control their weight would be negatively associated with energy balance-related behaviors during the pandemic. In contrast, we expected that boredom, stress, and food cravings would be positively associated with energy balance-related behaviors during the pandemic.

## Methods

### Study Design

The study design has been described in full detail elsewhere (11). Briefly, we conducted a cross-sectional study where a convenience sample of U.S. adults completed an online survey delivered using Qualtrics (Qualtrics^®^ Software Company Provo UT and Seattle WA). All participants provided online consent before proceeding to complete the questionnaire. The Institutional Review Board at San Diego State University approved the study.

### Participants

We recruited 1,779 men (43.38%) and women (56.62%) between the age of 18 and 75 years. Inclusion criteria included: (1) access to the internet, and (2) living in the U.S. The questionnaire was administered through Amazon Mechanical Turk (Mturk, © 2005–2018, Amazon Mechanical Turk, Inc., Seattle, WA) (*n* = 1,267), a web service that enables researchers to survey the target population across the US (32). MTurk's workforce tends to be younger, educated, underemployed, with an equal distribution of males and females, a high percentage of Caucasians and Asians, and household incomes below the average US population (33, 34). We also collected data via social media, email, and word of mouth (*n* = 511). With these recruitment methods, we not only targeted the general population but also targeted support groups with persons of higher education on Facebook and Twitter. These two recruitment methods allowed us to include data from a diverse population.

A small compensation ($1.66) was given to eligible participants completing the survey through Mturk. This amount was estimated based on the minimal amount required to complete a similar survey and in line with the median hourly wage earned by an MTurk responder. Participants recruited via social media, email, and word of mouth volunteered to complete the survey and did not receive any monetary compensation. Of note, while the participation using this recruitment method was completely voluntary, it is possible that the compensation offered to Mturk workers for completion of survey may have been a motivational factor for them to participate in our study.

Participant recruitment and data collection occurred during the 11 days from April 24th, 2020 to May 4th, 2020, while shelter-in-place guidelines were instituted across the US. Of the 1,779 participants who initially responded to the call to complete the questionnaire, 1,609 participants were included in the data analysis. Of the 170 people excluded from the analysis (MTurk *n* = 112, Self-promotion *n* = 58), *n* = 116 failed to complete any questions related to behavioral and psychological variables, or analysis, or complete any the questionnaire or answer essential questions, or failed to respond to more than 2 attention check questions. Four attention check questions and one subjective question that asked participants to type a response in a text box were included to ensure responses were not bots. To assess the quality of participant responses, we also asked them to type their height (inches) and weight (pounds) in a text box, and any biologically implausible responses were excluded. Participants with missing body mass index or biologically implausible body mass index of <15 or BMI ≥ 57 kg/m^2^, calculated from self-reported height and weight were also excluded.

### Questionnaire

The Qualtrics questionnaire included the following 7 item categories: demographics, weight behaviors, sleep, and other health behaviors, eating behaviors, physical activity behaviors, psychological factors, and food purchasing behaviors. Questions within these categories were aimed at understanding change in practices and beliefs during the COVID-19 shelter-at-home. Similar to other studies, we asked whether these practices “increased,” “decreased,” or “stayed the same” during the COVID-19 shelter-at-home (35, 36). Based on the Qualtrics recordings, participants completed the survey in ~25 min. Cronbach's alpha, a measure of internal consistency reliability with higher values suggesting higher reliability, is indicated for each scale measure where applicable.

### Measures

#### Eating Behavior Measures

Eating behaviors were determined by asking participants if their consumption of the following items increased, decreased, or remained the same during COVID-19 shelter-in-place: fruits (during meals), vegetables (during meals), caffeine, non-diet drinks (includes, Coke, Pepsi, flavored juice drinks, sports drinks, sweetened teas, coffee drinks, energy drinks, electrolyte replacement drinks), and diet soda and other diet drinks. To determine change in consumption of processed and ultra-processed foods, we presented a list of foods as described by the NOVA classification system (37). This system classifies all foods into 4 groups based on the extent and purpose of industrial processing as following: unprocessed foods, processed culinary ingredients, processed foods, and ultra-processed foods (37). NOVA is a food classification system most applied in the scientific literature to identify and define ultra-processed foods (38). Ultra-processed foods are described as pre-prepared ready-to-heat products including pies and pasta and pizza dishes; poultry and fish “nuggets” and “sticks,” sausages, burgers, hot dogs, and other reconstituted meat products; and powdered and packaged “instant” soups, noodles, and desserts. We also collected information on the change in the following snack foods: cake, cookie, ice-cream, other desserts; chips, popcorn, pretzels, and crackers; gummy snacks, fruit candy, sour gummy, or other fruity candies; fruit; vegetables; chocolate; yogurt/cheese. Change in consumption of take-out food and alcohol intake was also recorded. Since no validated tool is available collect information of perceptual change in dietary behavior, a validated tool was not used to collect this data.

We also collected information on the change in consumption of snack items (cake, cookies, ice-cream, other desserts; chips, popcorn, pretzels, and crackers; gummy snacks, fruit candy, sour gummy, or other fruity candies; fruits; vegetables; chocolate; yogurt/cheese). Change in consumption of restaurant/take-out/fast food/delivery food and alcohol intake was also recorded. We did not collect data on quantities consumed for the specific food items using the traditional methods of self-reported dietary data collection because they are prone to reporting errors and appears to underestimate energy and nutrient intake (39, 40).

#### Physical Activity and Sedentary Measures

Change in sitting, walking, moderate physical activity, and vigorous physical activity during the COVID-19 outbreak in their area were assessed using “I am doing more,” “I am doing the same,” and “I am doing less” options. Change in sedentary behaviors was determined by asking questions on change in time spent on watching television, social media, or other leisurely activities such as video games, computer, email etc. since COVID-19 outbreak. Given the lack of validated questionnaires to capture the perceptual change in behaviors, we developed and used face-valid items for both the physical activity and eating behavior measures. We intentionally wrote these items to target if the energy balance behaviors “increased,” “decreased,” or “remained the same” to capture self-reported change.

#### The Control of Eating Questionnaire (CoEQ)

The validated CoEQ comprised 21 items and included questions on general appetite and overall mood (independent of craving), frequency and intensity of general food craving, craving for specific foods (e.g., dairy, starchy, sweet, or non-sweet foods), and individuals' perceived control over resisting craved food items. Participants responded about their experience over the previous seven days. These items were assessed using a 10-point visual analog scale (VAS). Subscales created form the questionnaire were used to calculate scores for: craving control, craving for sweet foods, craving for savory foods, and positive mood (41) and their α's were 0.91, 0.73, 0.78, and 0.75, respectively.

#### Sleep Duration and Sleep Quality

To assess sleep duration, participants were asked to report the average number of hours spent sleeping per day since the COVID lockdown in their area. To quantify sleep quality, we used the Stanford Sleepiness Scale (42) to collect ratings on how sleepy participants felt after waking up in the morning since the COVID lockdown in their area. This scale uses a 7-point rating scale to quantify a participant's sleepiness at the moment, where 1 is labeled “Feeling active and vital; alert; wide awake” and 7 is labeled as “Almost in reverie; sleep onset soon; lost struggle to remain awake. Higher values indicate greater sleepiness.

#### Multidimensional State Boredom Scale

The Multidimensional state boredom scale (43) was used to collect information on boredom during the COVID lockdown. This scale uses eight items to assess boredom in the present moment on a scale of 1 (*strongly disagree)* to 7 (*strongly agree)*. However, to capture boredom during the pandemic, we reframed each item by adding the phrase “since the COVID lockdown in my area” at the end of each statement (e.g., Time is passing by slower than usual, since the COVID lockdown in my area). Higher score indicated higher boredom during the lockdown. The scale has been used in a similar manner by others to measure boredom during the pandemic (44). Internal consistency of the items was high (α = 0.91).

#### Stress

All participants reported report their current stress levels using a visual analog scale. The scale ranged from 1 through 10, with 1 = no stress at all and 10=highest stress possible.

#### Capacity for Self-Control Scale

The Capacity for Self-Control Scale (45) assesses individual differences in the ability to exercise three forms of general self-control: self-control by inhibition (i.e., the ability to override a pull toward goal-inconsistent behavior), by initiation (i.e., the ability to override a push toward goal-inconsistent behavior), and by continuation (i.e., the ability to continue initiation or inhibition as a self-control challenge in ongoing). The abbreviated measure consists of 9 items (3 items per subscale) scored on a five-point Likert scale, from 1 (*hardly ever*) to 5 (*nearly always*). Responses to the scale items were reverse scored as appropriate and averaged (α = 0.86). Higher score indicates greater capacity for self-control trait.

#### Implicit Theory of Weight Measure

The Implicit Theory of Weight Measure (29) assesses the degree of orientation toward incremental beliefs of weight (i.e., beliefs that body weight is malleable). The measure consists of 6 items scored on a seven-point Likert scale from 1 (*strongly agree)* to 7 (*strongly disagree*). Responses to the scale items were reverse scored as appropriate and averaged (α = 0.82); higher scores indicate a higher degree of entity beliefs (i.e., beliefs that body weight is not malleable).

### Data Analysis

SAS version 9.4 (Cary, NC) and MPlus version 8.0 with Mixture software (46) were used for statistical analysis, and significance was set two-tailed at *p* < 0.05. We then calculated scale intercorrelations between psychological and health risk/protective factors. We created four energy balance behavior scores reflecting positive energy balance using the items on the Qualtrics survey administered. Items used to estimate a high-sedentary behavior score included change in television watching, change in screen time, and change in sitting time. Items used to estimate a low-physical activity behavior score included change in walking time, change in vigorous physical activity, and change in moderate physical activity. A high-unhealthy eating behavior score was calculated using responses on the soda, processed foods snacks, ultra-processed foods, snacking on sweets, snacking on chips/salty foods, snacking on gummy/fruity candies, snacking on chocolate, drinking alcohol, and eating takeout/fast food. The low-healthy eating behavior score was calculated using responses on fruit and vegetable consumption as snacks or in general during meals. All behaviors included in development of a priori energy balance behavior scores have been extensively reported to contribute to positive energy balance or negative energy balance (see Supplementary Material).

For change in each behavior related to energy intake or energy expenditure, we assigned scores to responses “I am doing more,” “I am doing the same,” and “I am doing less” such that, 1 = healthy change, 2 = no change, and 3 = unhealthy change. The α's for high-sedentary behavior score, low-physical activity behavior score, high-unhealthy eating behavior score, and low-healthy eating behavior score were 0.54, 0.63, 0.74, and 0.86, respectively. Note that scores on low-physical activity behavior and low-healthy eating behavior were calculated such that higher scores reflected less physical activity and less fruit and vegetable consumption.

We first conducted ANOVAs to test differences of health-risk behaviors between demographic groups. We then calculated intercorrelations between energy balance behavior scores and health and psychological risk and protective factors. We then characterized item level changes (increased, decreased, or stayed the same) for each health/psychological risk factor (see Supplementary Material). We further conducted a Latent Profile Analysis (LPA) to identify and characterize patterns of health behavior change during the pandemic. LPA is a data-driven approach used to uncover relationships among individuals to create meaningful groups (or classes) of people based on the heterogeneity of their responses; these classes can then be characterized and compared to each other using important demographic, psychological, and behavioral factors (47). Classes of people determined by LPA have been used to describe distinct differences in cognition and behavior among people with regard to a variety of physical and mental health phenomena, such as alcohol use, sleep, occupational stress, resilience, coping strategies etc. (48–50). In the current work, we used LPA to reveal different classes of people's health-risk behaviors during the COVID-19 pandemic shelter-at-home. We then compared the classes on psychological, behavioral, and demographic qualities to provide comprehensive representations of various groups of people's characteristics, thoughts, and behaviors during the COVID-19 pandemic shelter-at-home. This analysis does not focus on the amount of change within one behavior but instead looks at patterns of change (i.e., increase, decrease, stays the same) across multiple behaviors.

## Results

### Risk Behaviors by Demographic Groups

ANOVAs were conducted to evaluate differences of risk behaviors between demographic groups. Participants' scores for four energy balance behavior scores are presented for each demographic variable in Table 1. Briefly, the score for increasing sedentary behavior was significantly higher among women (vs. men; *p* < 0.001), Asians (vs. White people, Black people, and people who identified as “other” racial category; *p* = 0.015), unmarried (vs. married; *p* < 0.001) participants, and younger (18–39 years old vs. 40+ years old; *p* < 0.001) participants. The score for low-physical activity was significantly higher among Asians (vs. White people, Black people, and people who identified as “other” racial category; *p* < 0.001), unmarried (vs. married; *p* < 0.001) people, and among people in the lowest annual income bracket (< $30,000 vs. $30,000+; *p* = 0.009). The score for high-unhealthy eating was significantly higher among women (vs. men) and people in the highest annual income bracket (> $90,000 vs. < $90,000; *p* = 0.027), while the score for low-healthy eating was significantly higher among White people (vs. Asian people, Black people, and people who identified as “other” racial category; *p* = 0.039).

**Table 1:** Scores for four energy balance behavior categories by demographic profile of participants.

	**High-sedentary behavior score mean (SD)**	**Group comparison**	**Low-physical activity score mean (SD)**	**Group comparison**	**High-unhealthy eating score mean (SD)**	**Group comparison**	**Low-healthy eating score mean (SD)**	**Group comparison**
**Sex**								
Males (*N* = 684)	2.42 (0.43)	*F*_1,1559_ = 28.65 *p < 0.001* η^2^ = 0.02 [0.01, 0.03]	2.13 (0.53)	*F*_1,1557_ = 0.38 *p = 0.538* η^2^ = 0.00 [0.00, 0.00]	1.97 (0.34)	*F*_1,1590_ = 24.55 *p < 0.001* η^2^ = 0.02 [0.01, 0.03]	1.97 (0.48)	*F*_1,1576_ = 1.65 *p = 0.20* η^2^ = 0.00 [0.00, 0.01]
Females (*N* = 875)	2.54 (0.43)		2.11 (0.58)		2.06 (0.38)		1.94 (0.55)	
**Race**								
White (*N* = 1,209)	2.47 (0.44)	*F*_3, 1556_ = 3.52 *p = 0.015* η^2^ = 0.01 [0.00, 0.02]	2.09 (0.55)	*F*_3, 1554_ = 10.88 *p < 0.001* η^2^ = 0.02 [0.01, 0.04]	2.03 (0.35)	*F*_3, 1587_ = 1.94 *p = 0.121* η^2^ = 0.00 [0.00, 0.01]	1.97 (0.50)	*F*_3, 1573_ = 2.80 *p = 0.039* η^2^ = 0.01 [0.00, 0.01]
Black (*N* = 109)	2.49 (0.42)		2.15 (0.54)		1.96 (0.42)		1.85 (0.52)	
Other (*N* = 81)	2.52 (0.39)		2.08 (0.58)		2.07 (0.42)		1.95 (0.54)	
Asian (*N* = 158)	2.59 (0.39)		2.36 (0.59)		1.99 (0.43)		1.88 (0.59)	
**Ethnicity**								
Hispanic (*N* = 173)	2.52 (0.42)	*F*_1,1553_ = 0.96 *p = 0.327* η^2^ = 0.00 [0.00, 0.01]	2.12 (0.58)	*F*_1,1551_ = 0.00 *p = 0.994* η^2^ = 0.00 [0.00, 0.00]	2.00 (0.41)	*F*_1,1584_ =1.16 *p = 0.283* η^2^ = 0.00 [0.00, 0.01]	1.91 (0.57)	*F*_1,1570_ = 1.22 *p = 0.269* η^2^ = 0.00 [0.00, 0.01]
Not Hispanic (*N* = 1,380)	2.49 (0.43)		2.12 (0.56)		2.03 (0.36)		1.96 (0.51)	
**Marital status**								
Married (*N* = 748)	2.44 (0.43)	*F*_1,1558_ = 18.33 *p < 0.001* η^2^ = 0.01 [0.00, 0.02]	2.05 (0.55)	*F*_1,1556_ = 26.45 *p < 0.001* η^2^ = 0.02 [0.01, 0.03]	2.02 (0.37)	*F*_1,1589_ = 0.97 *p = 0.325* η^2^ = 0.00 [0.00, 0.01]	1.95 (0.51)	*F*_1,1575_ = 0.00 *p = 0.973* η^2^ = 0.00 [0.00, 0.00]
Not married (*N* = 810)	2.53 (0.43)		2.19 (0.56)		2.03 (0.36)		1.95 (0.53)	
**Age**								
18–39 (*N* = 967)	2.53 (0.43)	*F*_2, 1558_ = 9.32 *p < 0.001* η^2^ = 0.01 [0.00, 0.02]	2.13 (0.57)	*F*_2, 1556_ = 1.21 *p = 0.300* η^2^ = 0.00 [0.00, 0.01]	2.03 (0.38)	*F*_2, 1589_ = 1.26 *p = 0.285* η^2^ = 0.00 [0.00, 0.01]	1.95 (0.54)	*F*_2, 1575_ = 0.37 *p = 0.691* η^2^ = 0.00 [0.00, 0.00]
40–64 (*N* = 531)	2.44 (0.43)		2.11 (0.55)		2.02 (0.34)		1.95 (0.48)	
>64 (*N* = 61)	2.34 (0.40)		2.02 (0.38)		1.96 (0.32)		2.01 (0.43)	
**Income**								
<30,000 (*N* = 290)	2.45 (0.43)	*F*_3, 1489_= 2.33 *p = 0.073* η^2^ = 0.00 [0.00, 0.01]	2.20 (0.53)	*F*_3, 1487_ = 3.88 *p = 0.009* η^2^ = 0.01 [0.00, 0.02]	2.01 (0.36)	*F*_3, 1518_ = 3.08 *p = 0.027* η^2^ = 0.01 [0.00, 0.01]	1.72 (0.56)	*F*_3, 1507_ = 0.99 *p = 0.397* η^2^ = 0.00 [0.00, 0.01]
30,000–59,999 (*N* = 420)	2.46 (0.43)		2.14 (0.55)		2.01 (0.36)		1.97 (0.52)	
60,000–89,999 (*N* = 329)	2.53 (0.43)		2.08 (0.57)		2.00 (0.39)		1.97 (0.51)	
>90,000 (*N* = 452)	2.50 (0.43)		2.07 (0.56)		2.07 (0.35)		1.93 (0.53)	

*All risk scores are calculated such that higher values = less healthy behavior (e.g., more sedentary time, less physical activity). All eta-squared values are presented with 95% CI*.

### Correlations Between Psychological and Health Risk/Protective Factors

Scale intercorrelations were calculated to highlight associations between psychological and health risk and health protective factors. Correlations are shown in Table 2. A high level of boredom was associated with lower self-control (*p* < 0.01), positive mood (*p* < 0.001), and control of cravings (*p* < 0.001) and with higher beliefs about weight control (*p* < 0.001), cravings for sweet and savory foods (*p*'s <0.001), sleepiness (*p* < 0.001), and stress (*p* < 0.001). Higher self-control was associated with lower beliefs about weight control (*p* < 0.001), cravings for sweet and savory foods (*p*'s <0.001), sleepiness (*p* < 0.001), and stress (*p* < 0.001) and with higher positive mood (*p* < 0.001) and control of cravings (*p* < 0.001).

**Table 2:** Correlations between psychological and health risk/protective factors.

	**Mean (SD)**	**1**	**2**	**3**	**4**	**5**	**6**	**7**	**8**	**9**	**10**	**11**
Boredom (1)	3.74 (1.53)	—										
Self-control (2)	3.39 (0.78)	−0.62^**^	—									
Beliefs about weight control (3)	2.61 (1.19)	0.22^***^	−0.21^***^	—								
Positive mood (4)	5.51 (1.97)	−0.57^***^	0.53^***^	−0.14^***^	—							
Control of cravings (5)	5.52 (2.46)	−0.39^***^	0.45^***^	−0.06^*^	0.25^***^	—						
Cravings for sweet foods (6)	4.19 (2.33)	0.33^***^	−0.32^***^	0.12^***^	−0.21^***^	−0.74^***^	—					
Cravings for savory foods (7)	4.49 (2.03)	0.33^***^	−0.28^***^	0.05	−0.16^***^	−0.65^***^	0.60^***^	—				
Sleepiness rating (8)	2.90 (1.45)	0.42^***^	−0.45^***^	0.05^*^	−0.53^***^	−0.27^***^	0.17^***^	0.17^***^	—			
Hours of sleep (9)	7.31 (1.45)	0.01	0.00	0.09^***^	0.13^***^	−0.05	0.06^*^	0.03	−0.08^**^	—		
Body mass index (10)	25.99 (5.95)	−0.02	−0.06^*^	−0.03	−0.01	−0.17^***^	0.07^**^	0.12^***^	0.05^*^	−0.11^***^	—	
Stress (11)	4.59 (2.50)	0.46^***^	−0.36^***^	0.19^***^	−0.63^***^	−0.28^***^	0.27^***^	0.23^***^	0.40^***^	−0.09^***^	0.03	—

* *p < 0.05*,

** *p < 0.01*,

*** *p < 0.001*.

### Latent Profile Analysis

**Figure 1:**
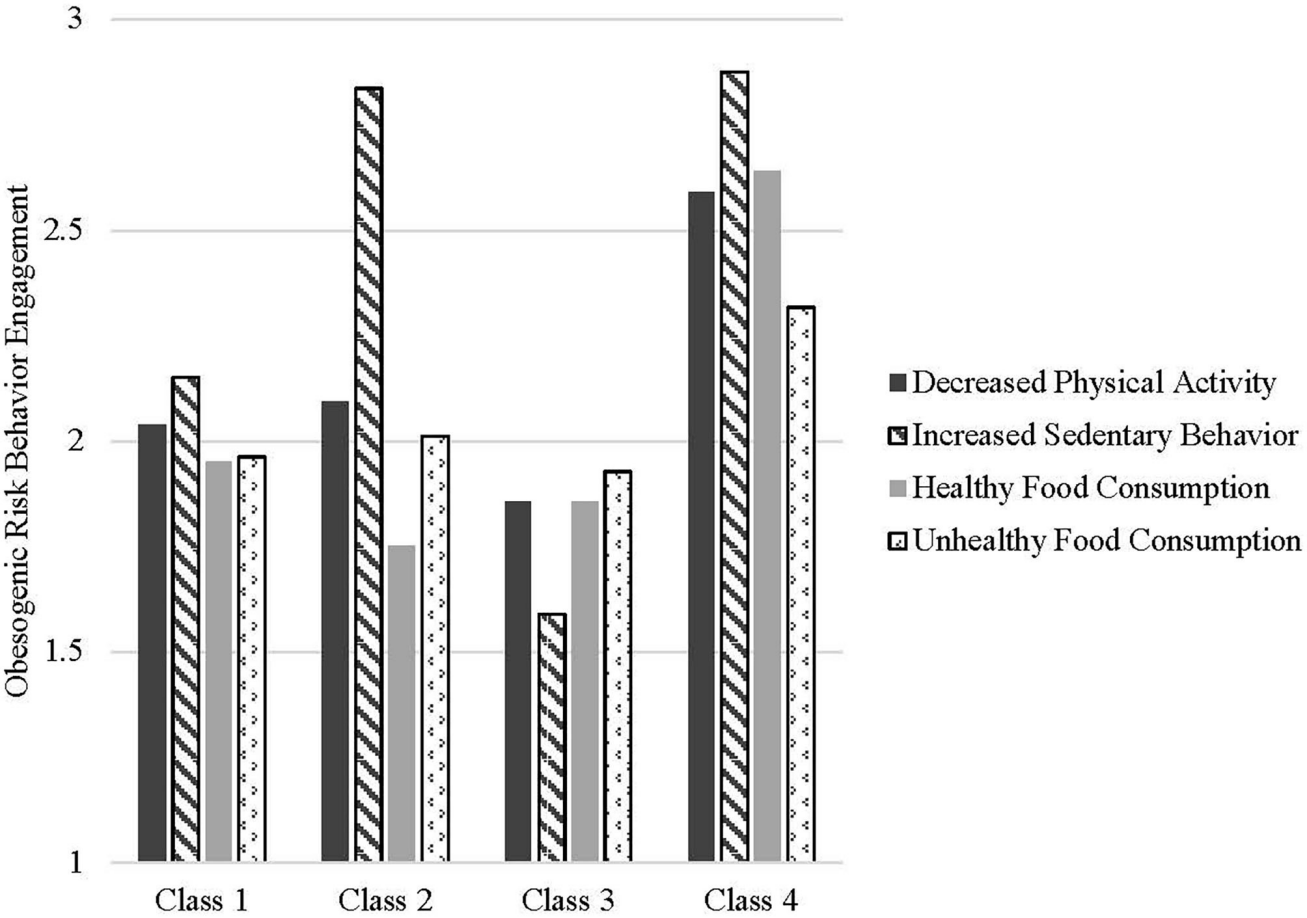
Average scores of engagement in obesogenic risk behaviors by latent classes. Class 3 is considered the General Low Risk Group; Class 4 is considered the General High-Risk Group. Class 1 is the Medium General Risk, Medium Sedentary Risk Group, and Class 2 is the Medium General Risk, High Sedentary Risk Group.

Next, we conducted a LPA to characterize classes of participants' patterns of risky health behaviors during the COVID-19 pandemic using composite variables for physical activity, sedentary behavior, healthy food consumption, and unhealthy food consumption. A model with four classes demonstrated the best fit with the data, Log Likelihood (LL) = −3744.75, degrees of freedom (df) = 23, Aikake Information Criterion (AIC) = 7535.49, Bayes Information Criterion (BIC) = 7659.10, Sample-size adjusted BIC (ABIC) = 7586.03, Entropy = 0.826. The classes' patterns of endorsed risky health behaviors are shown in Figure 1.

Examining the characteristics of participants in all risk profiles (Table 3), individuals in the highest risk class (Class 4; 10.6% of the sample) had the highest levels of risk across all four indices (*p* < 0.001). They also reported being sleepier upon waking up (*p* < 0.001), being more bored (*p* < 0.001), having less self-control (*p* < 0.001), having less positive mood (*p* < 0.001), and having more cravings for sweet/savory foods (*p* < 0.001). Participants in the low-risk category (Class 3; 5.02% of sample) were generally similar to the medium risk classes, with one exception–they reported having lesser beliefs about the role of personal effort in weight maintenance than did participants in other groups. Classes 1 (43.35% of the sample) and 2 (41.03% of the sample) both reported generally medium-to high risk with one key behavioral difference: Class 2 reported very high increases in sedentary behavior whereas people sorted into Class 1 were more likely to report engaging in about the same amount of sedentary behavior during the pandemic as before. In terms of psychosocial risk factors, Class 2 differed from Class 1 in sleep patterns (Class 2 participants reported waking up less alert despite reporting more hours of sleep), boredom, self-control, and mood. Although people in these classes were similar in physical activity and engaged in a mixed pattern of healthy and unhealthy eating habits, they exhibited different patterns of positive mood, craving control, cravings, boredom, and self-control. Demographic differences also emerged across groups. Participants in Classes 1 and 3 (relatively lower risk) were more likely to be male, married and White.

**Table 3:** Psychosocial risk factors across class determined by latent profile analysis.

	**Class 1 medium general risk, medium sedentary risk (*N* = 671–689)**	**Class 2 medium general risk, high sedentary risk (*N* = 643–654)**	**Class 3 general low risk (*N* = 80)**	**Class 4 general high risk (*N* = 165–169)**	**Comparison across class (Omnibus F)**	**η^2^ [95% CI]**
Low-physical activity score	2.04^a^	2.10^a^	1.89^b^	2.64^c^	63.85^***^	0.11 [0.08, 0.14]
High-sedentary behavior score	2.16^a^	2.84^b^	1.58^c^	2.89^d^	2918.05^***^	0.85 [0.84, 0.86]
High-unhealthy eating score	1.96^a^	2.02^b^	1.94^a^	2.33^c^	51.69^***^	0.09 [0.06, 0.12]
Low-healthy eating score	1.95^a^	1.74^b^	1.88^a^	2.80^c^	284.24^***^	0.35 [0.32, 0.38]
Boredom	3.38^a^	3.99^b^	3.33^a^	4.42^c^	32.52^***^	0.06 [0.04, 0.08]
Self-control	3.53^a^	3.35^b^	3.43^a^	2.94^c^	27.32^***^	0.05 [0.03, 0.07]
Beliefs about weight control	2.64^a^	2.57^a^	3.00^b^	2.46^a^	4.17^**^	0.01 [0.00, 0.02]
Positive mood	5.81^a^	5.44^b^	5.66^ab^	4.44^c^	21.81^***^	0.04 [0.02, 0.06]
Control of cravings	5.93^a^	5.43^b^	5.76^ab^	4.06^c^	26.21^***^	0.05 [0.03, 0.07]
Cravings for sweet foods	3.85^a^	4.35^b^	4.01^ab^	5.07^c^	13.68^***^	0.03 [0.01, 0.04]
Cravings for savory foods	4.12^a^	4.67^b^	4.34^ab^	5.48^c^	21.68^***^	0.04 [0.02, 0.06]
Sleepiness rating	2.65^a^	2.96^b^	2.78^ab^	3.68^c^	24.70^***^	0.04 [0.03, 0.06]
Hours of sleep	7.23^a^	7.41^b^	7.24^ab^	7.26^ab^	2.04	0.00 [0.00, 0.01]
Body mass index	26.20^a^	25.67^a^	25.59^a^	26.45^a^	1.38	0.00 [0.00, 0.01]
Stress	4.29^a^	4.70^b^	4.31^ab^	5.49^c^	11.61^***^	0.02 [0.01, 0.04]
**Demographics**					**Chi square comparison across group**	**Cramer's V**
Age	40.14^a^	36.31^b^	39.09^abc^	35.75^bc^	*F* = 12.17^***^	η^2^ = 0.02 [0.01, 0.04]
Male	51.23%	37.77%	43.75%	33.73%	32.12^***^	0.14
Married	51.66%	44.65%	57.70%	36.89%	15.34^**^	0.10
Hispanic	10.30%	12.40%	8.97%	11.31%	1.90	0.03
**Race**						
White	79.45%	74.92%	88.61%	72.78%	18.10^*^	0.06
Black	6.51%	7.80%	6.33%	5.92%		
Asian	8.54%	11.93%	5.06%	15.38%		
Other	5.50%	5.35%	0.00%	5.92%		
**Income**						
< $30,000	22.36%	17.71%	15.79%	16.36%	14.84	0.06
$30,000–59,999	29.00%	25.93%	34.21%	29.09%		
$60,000–89,999	20.85%	23.35%	14.47%	25.45%		
>$90,000	27.79%	33.01%	35.53%	29.09%		

* *p < 0.05*,

** *p < 0.01*,

*** *p < 0.001. Different superscript letters indicate statistical significance when testing between group differences*.

## Discussion

The primary purpose of this paper was to investigate the relationship between relevant psychological markers and energy balance-related behavior scores, during the COVID-19 related shelter-in-place. Generally, we report that increased boredom, higher self-reported cravings for sweet/savory foods, and high sleepiness ratings during the lockdown were related to increased unhealthy eating and sedentary behavior and decreasing physical activity and healthy eating during the lockdown. Whereas, having psychological traits such as greater general self-control, control over cravings, or positive mood was related to lower self-reported energy intake and energy expenditure during the lockdown. Individuals with the highest risk pattern reported having higher sleepiness, more boredom, less positive mood, and more cravings for sweet and savory foods.

Our hypothesis that self-reported change in boredom during the lockdown, a state like-psychological variable, may be related to dietary intake risk was based on prior research suggesting that high boredom increases the desire for and intake of unhealthy foods and snacks (23). Indeed, in a recent survey of French adults, 37–47% of respondents reported to increase eating to reduce stress, boredom, and feelings of emptiness experienced during the COVID-19 lockdown (51). Our data support these findings by showing that boredom was related to the increased risk of consuming unhealthy foods (energy-dense sweet and savory snacks, sugary drinks, etc.) and lowering healthy food intake (fruits and vegetables) during the pandemic. Boredom is shown to encourage people to seek sensation (52); hence, we speculate that exciting options, such as sugary and fatty foods, may have served as a potent distractor of self-regulation by providing intense appearance or taste. Another common reaction to boredom is to give up on a task because of decreased attention and/or greater perceived task difficulty (53). As a result, people gravitate toward easier tasks that require less cognitive load, such as the use of smartphones, the internet, or online socializing (54, 55); this may explain the relationship observed between increased sedentary behavior, low physical activity and boredom, in our dataset.

The relationship observed between self-reported sleepiness ratings, sleep duration, and diet quality in the current study confirms results from prior studies. We, and others, have previously shown that higher sleepiness (26, 56) and reduced sleep duration (57) are both related to food cravings and intake of energy-dense savory and sugary foods that may manifest in positive energy balance. The relationship of sleep time with sedentary activity is more complex, with long and short sleep durations both shown to impact sedentary behaviors in previous studies. In particular, reduced sleep duration (<7 h/night) correlates with increases in self-reported sitting minutes (58) and spending more time in front of the television (59), thus adding to sedentary time. In contrast, long sleep duration lowers daytime activity levels and increases screen-based sedentary behaviors (60). These data suggest that the reported positive correlation between sleep duration and sedentary activity is possibly related to a decline in overall wake time activity. We further speculate that lethargy after a long sleep duration and having less time available in the day may have added to increased sedentary behavior. It is equally possible that spending more sedentary time, especially in front of the screen, may reduce sleep quantity and quality (61). Given the cross-sectional design of this study, it is difficult to determine the directionality of the relationship between sleep duration and sedentary behavior in our participants during the shelter-at-home.

Similar to the findings by Buckland et al. where lower craving control predicted high energy dense sweet and savory food intake during COVID-19 lockdown, we also showed that greater control on food cravings, representing a state-like psychological characteristic, was related to unhealthy eating score (62). Intense food craving is often accompanied with lower mood and anxiety levels, and commonly reported with high BMI (63). Accordingly, we demonstrated that high craving control correlated with positive mood score and healthy food selection. Our data also shows a relationship between craving control and low reduction in physical activity. Interestingly, physical activity interventions can reduce cravings for high-caloric foods as well as mood (64). While we cannot confirm directionality in our cross-sectional data, it is possible that maintenance of high physical activity contributed to better mood and low boredom, thus supporting control over cravings.

In everyday life, general self-control, a trait psychological characteristic, is associated with positive weight management behaviors, including healthier eating, successful weight loss, and increased physical activity, as well as with better psychological well-being (65–67). The current study extends previous research on the personal benefits of self-control by highlighting the potentially protective aspects of self-control during a time when typical lifestyles have been majorly disrupted—in the context of a global pandemic. Because uncertainty increases the desire for indulgence (68), having high self-control may buffer temptation engagement during COVID-19 shelter-in-place. Notably, in this study, people who reported the least engagement in energy balance-related behaviors had the highest self-control. Those with relatively higher self-control also reported feeling in control of their food cravings, had fewer cravings for sweet and for savory foods, believed that body weight is malleable, and had lower average BMI. It could be that people who have higher self-control are better able to continue their established physical activity routines and habits of inhibiting unhealthy food consumption in times of uncertainty (69, 70) and to initiate new lifestyle adjustments in the face of necessary change (45). People with high self-control may also be adept at avoiding tempting situations (71, 72), which may happen frequently during shelter-in-place (e.g., ordering restaurant food to be delivered to one's house, watching more hours of television). In addition, people with higher self-control experienced several positive emotional benefits during shelter-in-place: on average, they felt less bored, reported higher positive mood, more alertness after waking, and less stress. Being able to successfully navigate temptation, resolve self-control conflicts, and pursue their goals, even in an unpredictable time, likely has a beneficial effect on mental well-being (66). Taken together, trait self-control may be a protective factor against the negative effects of COVID-19 shelter-in-place.

One predictor of weight management behaviors is the belief that a person's body weight is malleable (29–31, 73). In contrast to previous work, however, people in the current study who were classified as engaging the least in energy balance-related behaviors (vs. people in the higher risk classes) reported stronger beliefs that body weight is not malleable. Replicating previous correlational findings (30, 74), in the current study, participants' beliefs about weight malleability were unrelated to their BMI. Surprisingly, people who had stronger entity beliefs about body weight reported less sedentary behavior and less unhealthy eating; beliefs about weight control were unrelated to physical activity risk and healthy eating risk. One possible explanation for this finding might be that people who believed they can control their weight felt like they might be able to regain energy balance after the pandemic—that they could manage their weight well when they had the time and resources to do so. Counterintuitively, their health behaviors during the pandemic may have slipped because they thought they might be able to make up for it later. Alternatively, it may be that self-efficacy—which is a mechanism by which beliefs about weight control influence health behaviors (29, 74)—was interrupted during the COVID-19 pandemic. It could also be the case that during this unprecedented time, people may have generally low beliefs that if they were to experience setbacks in their weight management pursuits, they would be able to successfully cope with those challenges. Although we did not directly measure self-efficacy nor expectations of future success, people who reported having weaker incremental weight beliefs also reported lower positive mood, less control over their food cravings, higher cravings for sweet foods, less alertness after waking, and higher stress. Participants' negative mood may signal to them that they are making poor progress on their goals and will subsequently be less successful in the future (75), which may be indicative of their engagement with weight-management behaviors. In our study, people with more positive mood had a lower risk of less physical activity and unhealthy eating. Along the same lines, feelings of control of one's food cravings predict lower risks of unhealthy and healthy eating. These negative psychological factors experienced during shelter-in-place may attenuate the otherwise positive effect that incremental beliefs usually have on weight management behaviors.

Given the heterogeneity in energy balance-related behaviors, an assessment of risk profile groups gave us a better insight into the unique characteristics of individuals who may be more prone to weight gain during the pandemic. Not surprisingly, individuals with the highest risk not only engaged in all energy balance-related behaviors but also reported to have psychological and health markers known to promote obesity. Although similar in risk level, we observed subtle but unique differences between the two moderate risk groups. The most striking difference between the two groups was sedentary behavior. As theorized by previous work, a complex interplay between personal circumstances, environmental variables, and social factors determines sedentary behavior (76). A large percentage of high sedentary risk group (Class 2) individuals belonged to a high-income bracket. High income groups are more likely to hold sedentary jobs (77) and are known to engage in prolonged sedentary behavior, as compared to lower income groups. Occupational sitting and screen time, along with the closure of all outdoor avenues and added pressure of being *always on* when working from home, may have put the higher income group at higher risk. We also noticed that a large percentage of adults in this group were married or living with a partner. While we did not measure it directly, there is a plausibility of higher perceived modeling of sedentary behavior in presence of a partner, especially if the partner spends more time engaged in screen time (78). Additionally, perceived behavioral control is likely to be protective of sedentarism (79), which was prevalent in the Class 2 risk group. By contrast, studies also show that when it comes to sedentary behaviors, self-control beliefs may be ineffective in influencing the decision to be sedentary. Rather it is the discriminant motivational structure, high access, and ease of use among people who wish to perform these behaviors (80). This lack of motivation with high boredom and negative mood may have been the differentiating factor for sedentary behavior in the two groups during the pandemic.

The results of this study must be interpreted in light of several limitations. This study was cross-sectional and non-experimental; thus, causality and temporality cannot be inferred. As such, we cannot conclude if reported alterations in behaviors truly lead to weight gain. Additionally, while there is evidence of behavior changes with body mass index status, due to the self-reported nature of height and weight data collected, we did not test the difference in health behaviors between BMI groups. We also asked participants to report their perception of behavior change (increased, decreased, remained the same), rather than asking them to report behaviors before and during the lockdown period and calculating the change score for each variable. While we did this to minimize self-reporting bias and/or recall bias, the data is still self-reported, and our results may be subject to biases. Moreover, a recent report demonstrated that perceptual increase in physical activity is driven by the amount of vigorous physical activity performed, suggesting that an increase in intensive physical activity is important for perceiving a change in one's physical activity (81). In contrast, smaller changes may need to be sufficient for change to be perceived as such (82). Thus, the self-reported change scores in our study may not be accurate. Furthermore, with possible differences in perception of individual behavioral component of score categories, our aggregate scores for these categories may be subject to biases. While pandemic related restrictions limited our ability to collect data on energy balance behaviors subjectively, the importance of using objective measures cannot be denied. Recall bias, especially with using non-validated tools, may confound self-reports reflecting a *perceived* rather than *actual* change behaviors during the lockdown (83). This should be taken into consideration when interpreting our findings.

Additionally, while we did not disclose the specific purpose of the study to the participants, our results could also be driven by participant's expectation and not their actual behavior. With regards to the questionnaires, while validated instruments were used as possible, some necessary questions were developed by the investigators to capture the current unique environment. Moreover, we did not use a validated tool for dietary intake, such as food frequency questionnaires. Thus, care should be taken to integrate these findings with the broader literature. For our psychological and health behavior constructs, some variables were contextual or state like, while some were trait like. However, this should not have impacted our findings because whether it is a state like characteristic or trait like characteristic, we were interested in how it influenced energy-balance-related behaviors and how they differed between the risk classes. Moreover, despite the diversity and size of our sample, a convenience sampling approach was used, which may limit generalizability. Furthermore, the degree of shelter-in-place guidelines and the number of COVID-19 cases in participants' area of residence likely differed, creating differences in flexibility with stepping outside the house. The time frame of data collection may have influenced our results as well. As such, at the time of data collection, although most states had implemented shelter-in-place guidelines, a few states were considering lifting the restrictions. This one snapshot of time also assumes that thoughts and behaviors were static throughout the entire shelter-in-place time, which is likely an oversimplification.

Altogether, this study describes state- and trait-like psychological factors that relate to energy balance-related behavior categories during the COVID-19 shelter-at-home restrictions in the U.S. Our analysis provides important insights into the complex interplay of factors related to risk of increasing unhealthy eating and sedentary activities and decreasing healthy eating and physical activity. These results also contribute to improving our understanding of the patterns of risk groups and their unique characteristics, specifically highlighting that the lockdown did not adversely impact energy balance behaviors in all individuals. Our risk classes identified risk groups that represented 15–20% of our sample population. Health entities such as World Health Organization have several nutritional and lifestyle recommendations to follow during lockdown for the general public Thus, based on our findings, such public health efforts may be better spent targeting at-risk population subgroups in need of weight management interventions during the current pandemic rather than targeting people who are already managing the transition well. Our results also suggest that self-reported changes in state-like psychological variables impacted energy balance behaviors in a similar manner during COVID-19 lockdown, as they did during pre-COVID time. Thus, an effort to reduce stress and boredom, improve sleep hygiene, and strategies to control food cravings (all state-like psychological variables) using public health platforms may be beneficial in addressing a potential negative impact of lockdown on energy balance behaviors. Additional research is also needed on collecting longitudinal data to understand whether the high-risk behaviors revert back to normal as the pandemic crisis is passed.

## Data Availability Statement

The raw data supporting the conclusions of this article will be made available by the authors, without undue reservation.

## Ethics Statement

This study protocol (HS-2020-0105, HS-2020-0100) was reviewed and approved by the Institutional Review Board at San Diego State University, California. All participants gave an online informed consent before initiating the study questionnaire. The ethics committee waived the requirement of written informed consent for participation.

## Author Contributions

SB, JC, and MD conceived and designed the experiment and acquired the data. MD and LH analyzed the data. SB, JC, LH, and MD interpreted the results and wrote the paper. All authors contributed to the article and approved the submitted version.

## Conflict of Interest

The authors declare that the research was conducted in the absence of any commercial or financial relationships that could be construed as a potential conflict of interest.

## Abbreviations

BMI: Body mass index
Mturk: Amazon Mechanical Turk
CoEQ: The Control of Eating Questionnaire.
