# Supplementary material for: Energy Balance-Related Behavior Risk Pattern and Its Correlates During COVID-19 Related Home Confinement
Source: Front Nutr. 2021 Jun 8;8:680105. doi: 10.3389/fnut.2021.680105 (PMC8217605; doi:10.3389/fnut.2021.680105)
Supplement: Supplementary file 1 [file Data_Sheet_1.pdf]

Supplementary Tables

Supplementary Table 1. Energy intake and Energy Expenditure Behavior Components Used to Calculate Energy Balance Behavior Scores.

**Components included in calculation of energy balance behavior scores**
**Low-healthy eating behavior score components**
Vegetable Consumption
Fruit Consumption
Snacking on Fruit
Snacking on Vegetables
Snacking on Yogurt/Cheese
**High-unhealthy healthy eating behavior score components**
Non-diet drinks (all SSBs)
Diet Soda or other diet drinks
Processed foods
Ultra-processed foods
Snacking on desserts (Cake, cookie, ice-cream etc.)
Snacking on chips, popcorn, pretzels, and crackers
Snacking on Gummy/Fruity Candies
Snacking on Chocolate
Eating Takeout
**Low-physical activity behavior score components**
Vigorous PA
Moderate PA
Walking
**High-sedentary activity behavior score components**
Watching TV
Screen Time
Sitting

Supplementary Table 2. Self-Reported Energy Intake and Energy Expenditure Components with Change in Psychosocial Risk Factors

	Boredom				Self-Control				Cravings				Positive Mood			
	↑sed	Same	↓sed	F	↑sed	Same	↓sed	F	↑sed	Same	↓sed	F	↑sed	Same	↓sed	F
Vegetable Consumption	3.78^{a}	3.57^{b}	4.33^{c}	24.40***	3.38^{a}	3.48^{b}	3.02^{a}	35.94***	3.38^{a}	3.48^{b}	3.02^{a}	35.94***	2.68^{a}	2.61^{a}	2.57^{a}	1.12
Fruit Consumption	3.93^{a}	3.49^{b}	4.25^{c}	29.41***	3.31^{a}	3.51^{b}	3.07^{c}	35.79***	3.31^{a}	3.51^{b}	3.07^{c}	35.79***	2.68^{a}	2.54^{a}	2.67^{a}	2.53
Non-diet drinks (all SSBs)	4.22^{a}	3.63^{b}	4.29^{a}	23.08***	3.18^{a}	3.43^{b}	3.13^{a}	17.50***	3.18^{a}	3.43^{b}	3.13^{a}	17.50***	2.50^{a}	2.59^{a}	3.14^{b}	16.93***
Diet Soda or other diet drinks	4.33^{a}	3.58^{b}	4.14^{a}	21.49***	3.14^{a}	3.44^{b}	3.17^{a}	15.31***	3.14^{a}	3.44^{b}	3.17^{a}	15.31***	2.67^{a}	2.58^{a}	3.16^{b}	15.38***
Processed foods	4.17^{a}	3.47^{b}	3.96^{a}	35.15***	3.10^{a}	3.55^{b}	3.26^{c}	52.81***	3.10^{a}	3.55^{b}	3.26^{c}	52.81***	2.51^{a}	2.57^{a}	2.97^{b}	10.49***
Ultra-processed foods	4.28^{a}	3.49^{b}	3.97^{c}	38.44***	3.09^{a}	3.50^{b}	3.25^{c}	39.10***	3.09^{a}	3.50^{b}	3.25^{c}	39.10***	2.54^{a}	2.54^{a}	2.95^{b}	11.39***
Snacking on desserts (Cake, cookie, ice-cream etc.)	4.25^{a}	3.49^{b}	3.94^{c}	38.44***	3.08^{a}	3.52^{b}	3.28^{c}	48.71***	3.08^{a}	3.52^{b}	3.28^{c}	48.71***	2.55^{a}	2.64^{ab}	2.82^{bc}	3.47*
Snacking on chips, popcorn, pretzels, and crackers	4.23^{a}	3.42^{b}	3.97^{c}	47.71***	3.11^{a}	3.53^{b}	3.39^{c}	46.20***	3.11^{a}	3.53^{b}	3.39^{c}	46.20***	2.53^{a}	2.59^{a}	3.04^{b}	12.44***
Snacking on Gummy/Fruity Candies	4.23^{a}	3.63^{b}	3.99^{a}	13.23***	3.15^{a}	3.41^{b}	3.28^{a}	9.37***	3.15^{a}	3.41^{b}	3.28^{a}	9.37***	2.65^{a}	2.64a	2.96^{b}	5.20**
Snacking on Fruit	3.97^{a}	3.47^{b}	4.28^{c}	33.70***	3.34^{a}	3.51^{b}	3.04^{c}	36.45***	3.34^{a}	3.51^{b}	3.04^{c}	36.45***	2.67^{a}	2.67^{a}	2.56^{a}	1.42
Snacking on Vegetables	3.84^{a}	3.54^{b}	4.40^{c}	30.57***	3.41^{a}	3.47^{a}	3.00^{b}	35.36***	3.41^{a}	3.47^{a}	3.00^{b}	35.36***	2.77^{a}	2.56^{b}	2.61^{a}	3.44*
Snacking on Chocolate	4.23^{a}	3.53^{b}	3.99^{a}	27.95***	3.05^{a}	3.49^{b}	3.31^{c}	38.82***	3.05^{a}	3.49^{b}	3.31^{c}	38.82***	2.62^{a}	2.55^{a}	2.91^{b}	7.15***
Snacking on Yogurt/Cheese	4.19^{a}	3.57^{b}	4.17^{a}	22.69***	3.19^{a}	3.45^{b}	3.17^{a}	16.39***	3.19^{a}	3.45^{b}	3.17^{a}	16.39***	2.71^{a}	2.53^{b}	3.11^{c}	14.78***
Eating Takeout	4.04^{a}	3.55^{b}	3.78^{c}	7.16***	3.26^{a}	3.52^{b}	3.35^{a}	9.60***	3.26^{a}	3.52^{b}	3.35^{a}	9.60***	2.54^{a}	2.62^{a}	2.60^{a}	0.29
Consuming Calories	4.24^{a}	3.35^{b}	3.88^{c}	56.75***	3.11^{a}	3.57^{b}	3.39^{c}	57.26***	3.11^{a}	3.57^{b}	3.39^{c}	57.26***	2.59^{a}	2.58^{a}	2.71^{a}	1.27
Consuming Caffeine	4.24^{a}	3.53^{b}	4.08^{a}	32.41***	3.22^{a}	3.46^{b}	3.27^{a}	14.20***	3.22^{a}	3.46^{b}	3.27^{a}	14.20***	2.67^{a}	2.54^{a}	2.90^{b}	7.94***
Watching TV	4.06^{a}	3.36^{b}	3.80^{a}	39.58***	3.30^{a}	3.49^{b}	3.32^{a}	11.27***	3.30^{a}	3.49^{b}	3.32^{a}	11.27***	2.59^{a}	2.58^{b}	2.91^{a}	2.95
Screen Time	4.10^{a}	3.29^{b}	3.57^{b}	54.92***	3.25^{a}	3.55^{b}	3.50^{b}	29.60***	3.25^{a}	3.55^{b}	3.50^{b}	29.60***	2.56^{a}	2.63^{a}	2.90^{b}	3.00*
Vigorous PA	3.73^{a}	3.48^{b}	4.08^{c}	24.04***	3.41^{a}	3.50^{a}	3.23^{b}	18.73***	3.41^{a}	3.50^{a}	3.23^{b}	18.73***	2.70^{a}	2,59^{a}	2.57^{a}	1.27
Moderate PA	3.87^{a}	3.40^{b}	4.17^{c}	40.28***	3.43^{a}	3.52^{a}	3.17^{b}	31.66***	3.43^{a}	3.52^{a}	3.17^{b}	31.66***	2.84^{a}	2.52^{b}	2.60^{b}	7.21***
Walking	3.88^{a}	3.36^{b}	4.00^{a}	27.40***	3.33^{a}	3.58^{b}	3.24^{a}	27.21***	3.33^{a}	3.58^{b}	3.24^{a}	27.21***	2.60^{a}	2.57^{a}	2.63^{a}	0.29
Sitting	4.05^{a}	3.25^{b}	3.61^{c}	49.67***	3.27^{a}	3.58^{b}	3.35^{a}	28.08***	3.27^{a}	3.58^{b}	3.35^{a}	28.08***	2.56^{a}	2.63^{b}	2.90^{c}	3.32*

*p<.05, **p<.01, ***p<.001. SSB: Sugar sweetened beverages

Supplementary Table 3. Self-Reported Energy Intake and Energy Expenditure Components with Change in Psychosocial Risk Factors

	Sleepiness				Hours of Sleep				BMI				Stress			
	**↑sed**	**Same**	**↓sed**	**F**	**↑sed**	**Same**	**↓sed**	**F**	**↑sed**	**Same**	**↓sed**	**F**	**↑sed**	**Same**	**↓sed**	**F**
Vegetable Consumption	2.79[a]	2.79[a]	3.46[b]	23.79***	7.49[a]	7.26[b]	7.16[b]	4.45*	26.07[a]	25.97[a]	26.17[a]	0.13	4.55[a]	4.36[b]	5.48[b]	20.56***
Fruit Consumption	2.83[a]	2.76[a]	4.43[b]	23.88***	7.52[a]	7.22[b]	7.29[b]	5.54**	25.89[a]	25.90[a]	26.25[a]	0.40	4.68[a]	4.35[b]	5.22[c]	13.25***
Non-diet drinks (all SSBs)	3.32[a]	2.84[b]	3.00[ab]	9.62***	7.25[a]	7.25[a]	7.66[b]	5.88**	26.68[a]	25.95[a]	25.98[a]	1.32	5.16[a]	4.47[b]	5.05[b]	9.18***
Diet Soda or other diet drinks	3.17[a]	2.83[b]	2.76[b]	3.79*	7.39[a]	7.26[ab]	7.59[b]	4.10*	27.24[a]	26.10[b]	26.36[ab]	2.17	5.31[a]	4.47[b]	4.64[b]	6.78**
Processed foods	3.28[a]	2.71[b]	2.80[b]	25.17***	7.21[a]	7.34[a]	7.36[a]	1.46	26.64[a]	25.88[b]	25.41[b]	3.73*	5.19[a]	4.20[b]	4.77[c]	25.05***
Ultra-processed foods	3.43[a]	2.72[b]	2.85[b]	31.92***	7.17[a]	7.33[ab]	7.49[b]	3.84*	26.63[a]	26.05[a]	25.11[b]	4.57*	5.36[a]	4.24[b]	4.74[c]	26.99***
Snacking on desserts (Cake, cookie, ice-cream etc.)	3.23[a]	2.73[b]	2.88[b]	17.12***	7.33[a]	7.35[a]	7.24[a]	0.47	26.70[a]	25.78[b]	25.90[ab]	3.42*	5.25[a]	4.28[b]	4.66[b]	22.37***
Snacking on chips, popcorn, pretzels, and crackers	3.33[a]	2.71[b]	2.70[b]	33.07***	7.29[a]	7.26[a]	7.43[a]	1.04	26.51[a]	26.00[a]	26.66[a]	1.80	5.13[a]	4.28[b]	4.67[b]	18.50***
Snacking on Gummy/Fruity Candies	3.12[a]	2.86[b]	2.88[ab]	2.34	7.36[a]	7.35[a]	7.35[a]	0.00	26.43[a]	26.02[a]	26.41[a]	0.53	5.18[a]	4.52[b]	4.56[b]	5.01**
Snacking on Fruit	2.86[a]	2.75[a]	3.44[b]	22.48***	7.48[a]	7.23[b]	7.20[b]	5.12**	25.95[a]	26.03[a]	26.29[a]	0.26	4.76[a]	4.33[b]	5.18[c]	12.65***
Snacking on Vegetables	2.71[a]	2.79[a]	3.45[b]	22.19***	7.43[a]	7.25[a]	7.34[a]	1.89	25.85[a]	26.08[a]	26.38[a]	0.51	4.55[a]	4.34[a]	5.48[b]	19.39***
Snacking on Chocolate	3.19[a]	2.80[b]	2.88[b]	8.47***	7.29[a]	7.29[a]	7.33[a]	0.08	26.79[a]	25.93[b]	25.84[b]	2.66	5.26[a]	4.36[b]	4.54[b]	15.88***
Snacking on Yogurt/Cheese	2.98[ab]	2.82[a]	3.12[b]	3.34*	7.40[a]	7.27[a]	7.23[a]	1.09	26.16[a]	26.08[a]	25.91[a]	0.08	5.02[a]	4.39[b]	5.02[a]	8.70***
Eating Takeout	3.08[a]	2.86[a]	2.88[a]	1.66	7.37[a]	7.23[a]	7.34[a]	1.16	26.92[a]	25.75[b]	25.96[b]	2.63	5.05[a]	4.35[b]	4.61[a]	5.24**
Consuming Calories	3.22[a]	2.70[b]	2.83[b]	21.73***	7.29[a]	7.24[a]	7.54[b]	4.35*	27.04[a]	25.45[b]	25.69[b]	11.90***	5.14[a]	4.15[b]	4.78[a]	26.27***
Consuming Caffeine	3.20[a]	2.78[b]	2.99[ab]	10.70***	7.09[a]	7.31[b]	7.61[c]	8.21***	26.29[a]	25.88[a]	26.23[a]	0.77	5.23[a]	4.35[b]	4.83[a]	16.76***
Watching TV	3.00[a]	2.77[b]	2.92[ab]	4.73**	7.40[a]	7.18[b]	7.15[b]	4.76**	26.06[a]	25.94[a]	26.47[a]	0.31	4.82[a]	4.26[b]	4.76[ab]	9.59***
Screen Time	3.09[a]	2.61[b]	3.00[a]	21.47***	7.38[a]	7.19[b]	7.14[ab]	3.89*	25.70[a]	26.39[b]	26.55[ab]	2.78	4.90[a]	4.07[b]	5.12[a]	22.08***
Vigorous PA	2.70[a]	2.74[a]	3.19[b]	18.66***	7.49[a]	7.29[b]	7.22[b]	3.79*	25.27[a]	26.24[b]	26.14[b]	2.91	4.71[a]	4.23[b]	4.93[a]	12.64***
Moderate PA	2.74[a]	2.71[a]	3.24[b]	23.04***	7.54[a]	7.24[b]	7.27[b]	4.65***	25.05[a]	26.30[b]	26.12[b]	4.52*	4.78[a]	4.13[b]	5.12[a]	25.75***
Walking	2.84[a]	2.66[a]	3.18[b]	18.91***	7.43[a]	7.15[b]	7.32[ab]	5.01***	25.96[a]	26.33[a]	25.71[a]	1.50	4.73[a]	4.10[b]	4.91[a]	15.79***
Sitting	3.11[a]	2.57[b]	2.60[b]	36.50***	7.31[a]	7.24[a]	7.26[a]	0.30	25.85[a]	26.46[a]	25.18[a]	2.79	4.90[a]	3.99[b]	4.64[a]	23.67***

*p<.05, **p<.01, ***p<.001. SSB: Sugar sweetened beverages
